# Comparison of pancreatojejunostomy techniques in patients with a soft pancreas: Kakita anastomosis and Blumgart anastomosis

**DOI:** 10.1186/s12893-018-0420-5

**Published:** 2018-10-24

**Authors:** Shoji Kawakatsu, Yosuke Inoue, Yoshihiro Mise, Takeaki Ishizawa, Hiromichi Ito, Yu Takahashi, Akio Saiura

**Affiliations:** Department of Gastroenterological Surgery, Cancer Institute Hospital, Japanese Foundation for Cancer Research, 3-10-6 Ariake, Koto-ku, Tokyo, 135-8550 Japan

**Keywords:** Pancreatoduodenectomy, Pancreatojejunostomy, Pancreatic fistula

## Abstract

**Background:**

Postoperative pancreatic fistula (PF) is the main cause of operative mortality in patients who undergo pancreatoduodenectomy. Various pancreatoenteric anastomosis techniques have been reported to minimize the postoperative PF rate. However, the optimal method remains unknown. This study was performed to clarify the impact of pancreatojejunostomy on clinically relevant PF (CR-PF) between Blumgart anastomosis and Kakita anastomosis in patients with a soft pancreas.

**Methods:**

In total, 620 consecutive patients underwent pancreatoduodenectomy at our institute from January 2010 to December 2016, and 282 patients with a soft pancreas were analyzed (Blumgart anastomosis, *n* = 110; Kakita anastomosis, *n* = 176). Short-term outcomes were assessed, and univariate and multivariate analyses of several clinicopathological variables were performed to analyze factors affecting the incidence of CR-PF.

**Results:**

The CR-PF rate was 42.7% (122/286). The CR-PF rate was not significantly different between the Blumgart and Kakita groups (42.7% and 42.6%, respectively; *p* = 0.985). The morbidity rate (Clavien–Dindo grade ≥ IIIa) was 24.5% (70/286), and the operation-related mortality rate was 0.7% (2/286). In the multivariate analysis, male sex (*p* = 0.0245) and a body mass index of ≥22 kg/m^2^ (*p* < 0.0001) were statistically significant risk factors for CR-PF.

**Conclusions:**

The CR-PF rate was not significantly different between patients treated with Kakita versus Blumgart anastomosis.

## Background

Recent advances in surgical techniques and perioperative management have made it possible to reduce the postoperative mortality rate after pancreatoduodenectomy. A nationwide survey from Japan reported that the mortality rate after pancreatoduodenectomy was 2.9% [1]. The recently reported mortality rate after pancreatoduodenectomy in the US was 1.4% [2]. However, pancreatoduodenectomy remains a complex and technically demanding procedure, and postoperative pancreatic fistula (PF) is an unsolved problem. Most cases of mortality after pancreatoduodenectomy result from the development of postoperative PF, such as septic complications or intra-abdominal hemorrhage [3] from ruptured aneurysms. Although numerous pancreatoduodenectomy techniques have been proposed, there is no standardized procedure for preventing postoperative PF, especially in patients with a soft pancreas.

To minimize the incidence of postoperative PF, which is closely associated with subsequent mortality, we have contrived various pancreatoenteric anastomosis techniques, and several methods of pancreatojejunostomy (PJ) have been proposed in the literature. Among them, Kakita anastomosis, originally described by Kakita et al. [4] in 1996, is one of the most widely accepted procedures for PJ in Japan. In recent decades, a new standardized U-suture technique, which was originally described by Blumgart et al. [5, 6] in 2000, has been improved and rapidly accepted. Several studies have demonstrated the superiority of Blumgart anastomosis over Kakita anastomosis [7, 8].

Based on these reports, we hypothesized that the purse-string–like suture used in Blumgart anastomosis would be superior to Kakita anastomosis in achieving a surer water-tight anastomosis and lower incidence of PF, although such a suture might cause ischemic change of the pancreatic stump and a higher rate of latent PF. Beginning in July 2014, we changed the PJ method from modified Kakita anastomosis to modified Blumgart anastomosis in a phased manner. A soft pancreas texture was recently reported to be the most influential factor for postoperative PF [9–12]. From January 2010 to June 2014, the clinically relevant PF (CR-PF) rate after pancreatoduodenectomy reconstructed with Kakita anastomosis at our institute was 44.7% (76/170) among patients with a soft pancreas and 7.2% (11/152) among those with a hard pancreas. In the present large-scale retrospective cohort study, we analyzed the incidence of CR-PF between Kakita and Blumgart anastomosis for patients with a soft pancreas.

## Methods

### Patient selection

**Fig. 1:**
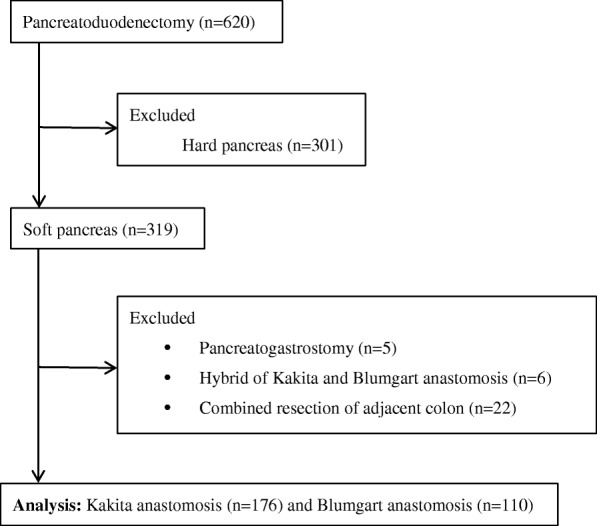
Flowchart of patient allocation

From January 2010 to December 2016, 620 consecutive patients underwent pancreatoduodenectomy at the Department of Gastroenterological Surgery, Cancer Institute Hospital, Japanese Foundation for Cancer Research, Tokyo, Japan. The institutional review board approved this study protocol. Among the 620 patients, 319 with a soft pancreas texture were enrolled in this study. Five patients who underwent pancreatogastrostomy and six who underwent a combination of Kakita and Blumgart PJ were excluded. Twenty-two patients who underwent concomitant resection of the adjacent colon were also excluded. In total, 286 patients were analyzed. Patient allocation in this study is summarized in Fig. 1.

### Surgical procedure

#### Resection

We basically performed subtotal stomach-preserving pancreatoduodenectomy. Systematic mesopancreas dissection using a supracolic anterior artery-first approach was performed as previously reported [13]. Before pancreas transection, the proximal side of the pancreas was ligated with 2–0 polyglactin, and the distal side was gently clamped by an intestinal forceps to control bleeding from the pancreatic stump. The method of pancreas transection was left to the surgeon’s discretion, and various methods were employed, such as the clamp-crushing method [14] or transection by a scalpel, ultrasonically activated device, or stapler.

#### Reconstruction

**Fig. 2:**
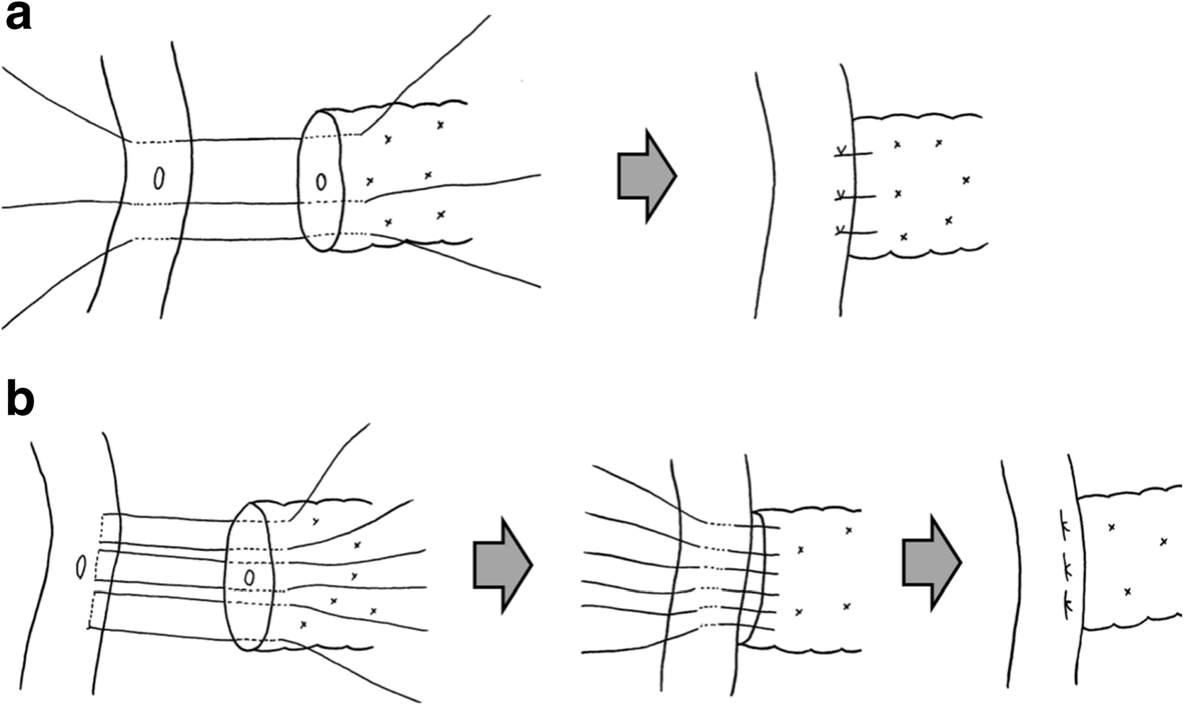
Pancreatojejunostomy method. Eight interrupted sutures with single-armed 6–0 polydioxanone for anastomosis of the main pancreatic duct to the jejunal mucosal layer (omitted from this schema) and several interrupted sutures with double-armed 3–0 polydioxanone for anastomosis of the pancreatic parenchyma to the jejunal seromuscular layer [(**a**) modified Kakita anastomosis or (**b**) modified Blumgart anastomosis]

Reconstruction was performed according to the modified Child’s technique. After the jejunal limb was brought up through the retrocolic root, PJ (8 interrupted sutures with single-armed 6–0 polydioxanone for anastomosis of the main pancreatic duct to the jejunal mucosal layer and several interrupted sutures with double-armed 3–0 polydioxanone for anastomosis of the pancreatic parenchyma to the jejunal seromuscular layer [modified Kakita anastomosis (Fig. 2a) or modified Blumgart anastomosis (Fig. 2b)]) was performed about 15 cm away from the end of the jejunal limb. An external drainage tube was inserted into the main pancreatic duct and brought out through the jejunal limb and abdominal wall. Choledochojejunostomy was then performed with 5–0 polyglyconate suture (interrupted sutures on the posterior wall and a running suture on the anterior wall) about 10 cm distal to the PJ. An external drainage tube was also inserted into the intrahepatic duct and brought out through the jejunal limb and abdominal wall. Gastrojejunostomy was then performed with a stapling device, and the insertion hole was closed with a hand-sewn Albert–Lembert suture (a running Albert suture with 4–0 polydioxanone and interrupted Lembert sutures with 4–0 polyglactin 910) about 40 cm distal to the choledochojejunostomy. Braun anastomosis was added with a 4–0 polydioxanone running suture. A feeding tube was routinely inserted into the jejunum. The round ligament was mobilized and wound around the stump of the gastroduodenal artery. Silicone drains with a diameter of 8 mm were routinely placed at the foramen of Winslow and the superior sides of the PJ in patients with a soft pancreas.

#### Modified Kakita anastomosis (Fig. 2a)

The parenchyma of the remnant pancreas was fixed to the jejunal seromuscular layer with two or three double-armed 3–0 polydioxanone penetrating sutures using gentle force to prevent laceration of the pancreatic parenchyma. The knots were placed on the jejunal serosa.

#### Modified Blumgart anastomosis (Fig. 2b)

The parenchyma of the remnant pancreas was fixed to the jejunal seromuscular layer with two or three double-armed 3–0 polydioxanone horizontal mattress sutures. One of the sutures strode across the main pancreatic duct to bind it.

### Definition of PF

Postoperative PF was diagnosed and graded in accordance with the International Study Group on Pancreatic Fistula classification [15]. PF was diagnosed when the amylase concentration in the drainage fluid on postoperative day 3 was more than three times the upper limit of the normal serum level. PF with an elevated inflammatory response on the blood examination and intravenous administration of antibiotics was defined as Grade B PF caused by infection. PF that required drain placement for > 22 days without an elevated inflammatory response or administration of antibiotics was defined as Grade B PF caused by long drain placement. Latent PF [16] was defined as PF that initially lacked amylase-rich effluent but ultimately progressed to CR-PF.

### Management of drainage tube

The amylase concentration of the drainage fluid was measured every day. When PF was evident, the drainage tube was exchanged on postoperative day 7, maintained with regular exchange until the drainage tube tract matured, and removed after the drainage fluid had nearly disappeared. In patients without postoperative PF, the drainage tube placed through the foramen of Winslow was removed on postoperative day 4, and the tube on the superior side of the PJ was removed on postoperative day 5.

### Analysis

All clinical data in the medical records were retrospectively reviewed. In this study, two major issues were analyzed using these data. First, short-term outcomes were compared among the patients who underwent Kakita anastomosis (Kakita group) and those who underwent Blumgart anastomosis (Blumgart group). Subgroup analyses of risk-stratified patients were also performed for CR-PF. Second, univariate and multivariate analyses of several clinicopathological variables were performed to analyze factors affecting the incidence of CR-PF. The texture of the pancreatic parenchyma was assessed by the operator’s palpation. The size of the main pancreatic duct was measured at the presumed surgical transection line on preoperative contrast-enhanced computed tomography.

### Statistical analysis

All statistical analyses were performed using JMP software version 10.0.2 (SAS Institute, Cary, NC). Categorical variables were analyzed using Pearson’s chi-square test or Fisher’s exact test as appropriate. Continuous variables were compared using the Mann–Whitney U test. Continuous data are presented as a range of median values. To identify prognostic factors in the study population, the clinicopathological variables were analyzed in a univariate proportional hazard model, and all variables associated with survival with a *p* value of < 0.1 were subsequently entered into a Cox multivariate regression model. Values of *p* < 0.05 were considered statistically significant.

## Results

### Baseline characteristics and short-term outcomes

Table 1 summarizes the baseline characteristics of the 286 patients and their short-term outcomes. The CR-PF rate was 42.7% (122/286). A drain placement duration of ≥22 days was the most common cause of Grade B PF, accounting for 48.4% (59/122) of cases. Twelve patients (4.2%) developed Grade C PF; reoperation was required for 6 patients, and arterial embolization for intra-abdominal bleeding was required for 6 patients. The median length of drain placement was 18.5 (4–127) days. The median postoperative hospital stay was 30 (8–127) days. The morbidity rate (Clavien–Dindo grade ≥ IIIa) was 24.5% (70/286), and the operation-related mortality rate was 0.7% (2/286). The readmission rate within 30 days after discharge and 90 days after the operation was 4.9% (14/286) and 7.0% (20/286), respectively. The most common reason for readmission was cholangitis (12/20 readmissions). Only one patient required readmission because of PF; this patient develop a pseudoaneurysm after conservative treatment for PF. Among the patients with operation-related mortality, one died of liver failure caused by postoperative bleeding arising from the PF and another died of aspiration pneumonia without development of PF.

**Table 1:** Patients characteristics and short-term outcomes

Variables	Total (*n* = 286)	Kakita (*n* = 176)	Blumgart (*n* = 110)	*p*
Patients characteristics				
Age (years)	67 (21–87)	66 (32–87)	69 (21–86)	0.143
Male	166 (58.0)	100 (56.8)	66 (60.0)	0.594
BMI	22.3 (15.9–32.0)	22.3 (15.9–32.0)	22.2 (16.1–31.6)	0.560
History of DM	47 (16.4)	27 (15.3)	20 (18.2)	0.530
Diameter of MPD (mm)	3 (1–16)	2 (1–8)	3 (1–16)	*0.0001* ^*^
Thickness of the pancreas (mm)	10 (5–18)	10 (5–18)	10 (6–18)	0.203
Shor-term outcomes				
Operative time (min)	481 (254–920)	487 (295–834)	477 (254–920)	0.782
Blood loss (mL)	450 (20–3530)	400 (20–3530)	490 (60–1875)	0.551
Pancreatic fistula (> Grade B)	122 (42.7)	75 (42.6)	47 (42.7)	0.985
Grade B	110 (38.5)	69	41	0.744
Length of drain placement > 22	59 (20.6)	39	20	0.416
Infection	51 (17.8)	30	21	0.661
Grade C	12 (4.2)	6 (3.4)	6 (5.5)	0.407
Re-operation	6 (2.1)	4	2	0.792
Intraabdominal bleeding (IAB)	4 (1.4)	2	2	0.637
Leakage of pancreatojejunostomy	2 (0.7)	2	0	0.163
Arterial embolization for IAB	6 (2.1)	2	4	0.158
Latent pancreatic fistula (> Grade B)	17 (5.9)	14 (8.0)	3 (2.7)	0.055
Morbidity (> Clavien-Dindo Grade IIIa)	70 (24.5)	45 (25.6)	25 (22.7)	0.586
Postoperative hospital stay	30 (16–127)	30 (16–108)	31 (16–127)	0.290
Length of drain placement	18.5 (4–127)	18 (4–85)	19 (4–127)	0.204
Mortality	2 (0.7)	1 (0.5)	1 (0.9)	0.740

All the data are shown as median (range) or the number (percentage)

^*^Indicates statistically significant

### Comparison between Kakita and Blumgart groups

Table 1 also compares the baseline characteristics and surgical outcomes between the Kakita and Blumgart groups. There was no significant difference in short-term outcomes, such as the incidence of CR-PF and latent PF, between the Kakita group (*n* = 176) and the Blumgart group (*n* = 110). The diameter of the main pancreatic duct was significantly larger in the Blumgart group.

### Univariate and multivariate analysis of clinicopathological variables

The results of the univariate and multivariate analyses of clinicopathological variables are shown in Table 2. The multivariate analysis showed that male sex (*p* = 0.0245) and a body mass index (BMI) of ≥22 kg/m^2^ (*p* < 0.0001) were statistically significant risk factors for CR-PF. There was no significant difference in the incidence of CR-PF between the Kakita and Blumgart groups.

**Table 2:** Univariate and multivariate analysis for risk factors of CR-PF

Variable	Univariate analysis			Multivariate analysis		
	OR	95% CI	*P*	OR	95% CI	*P*
Age > 70	1.40	0.87–2.25	0.1693			
Male	2.35	1.45–3.88	*0.0005* ^*^	1.90	1.12-3.23	*0.0170* ^*^
BMI > 22	3.75	2.28–6.26	*< 0.0001* ^*^	2.85	1.69–4.88	*< 0.0001* ^*^
Disease (pancreatic cancer)	1.19	0.69–2.03	0.5295			
History of DM	1.66	0.89–3.14	0.1121			
Pancreatojejunostomy (Blumgart)	1.00	0.62–1.63	0.9849	1.05	0.62–1.80	0.8465
Portal vein resection	1.14	0.59–2.18	0.6840			
SMD level (III)	1.41	0.71–2.80	0.3279			
Diameter of MPD > 3 mm	0.52	0.30–0.88	*0.0144* ^*^	0.57	0.32-1.01	0.0543
Thickness of the pancreas > 10 mm	1.32	0.82–2.12	0.2522			
Operative time > 500 min	1.88	1.17–3.04	*0.0096* ^*^	1.15	0.66-1.98	0.6184
Blood loss > 500 mL	2.48	1.54–4.04	*0.0002* ^*^	1.58	0.91-2.76	0.1043

*OR* Odds ratio, *CI* Confidence interval, *SMD* Systemic mesopancres dissection

^*^Indicates statistically significant

### Risk-stratified subgroup analysis of CR-PF between the Kakita and Blumgart groups

A subgroup analysis of high-risk subsets for CR-PF (age of ≥70 years, male, BMI of ≥22 kg/m^2^, main pancreatic duct diameter of ≤3 mm, and pancreatic thickness of ≥10 mm) as estimated by univariate and multivariate analyses was performed between the groups (Table 3). There were no significant differences in the rate of CR-PF between the two groups.

**Table 3:** Risk-stratified subgroup analysis of clinically relevant pancreatic fistula between Kakita and Blumgart groups

Subgroup	Kakita	Blumgart	*p*
Age of ≥70 years	51.5% (34/66)	42.3% (22/52)	0.3195
Male sex	53.0% (53/100)	48.5% (32/66)	0.5690
Body mass index of ≥22 kg/m^2^	53.1% (51/96)	62.7% (37/59)	0.2406
Main pancreatic duct diameter of ≤3 mm	44.7% (59/132)	52.2% (36/69)	0.3135
Pancreatic thickness of > 10 mm	53.9% (35/65)	40.8% (20/49)	0.1673

## Discussion

Several attempts to reduce the incidence of postoperative PF have been made in recent years, but no standard methods with which to minimize the incidence of postoperative PF have yet been established. According to a recent study, a soft pancreas texture is probably the most influential factor for postoperative PF [9–12]. In the current study, we compared the rate of CR-PF between the Kakita and Blumgart anastomosis groups of patients with a soft pancreas texture.

As shown in previous reports, male sex and a BMI of > 22 kg/m^2^ were risk factors for CR-PF [17] in our study. Unlike in previous reports [7, 8, 18], there was no significant difference in the incidence of CR-PF between the Kakita and Blumgart anastomosis groups. Our hypothesis that Blumgart anastomosis is associated with a lower incidence of whole PF and higher incidence of latent PF was denied in this study. In Blumgart anastomosis, the use of transpancreatic, full-thickness, mattress U-sutures instead of tangential sutures reportedly eliminates tangential tension and shear force at the stitch points of the pancreatic parenchyma because the pancreatic stump and stitch points are theoretically coved by jejunal serosa [18]. In Kakita anastomosis, the tangential suture through the pancreatic capsule may result in the development of shear forces at the stitch points of the pancreatic parenchyma, and more careful ligation is required. However, it is possible to completely cover the pancreatic cut end with jejunal serosa and protect the knots from cutting through the pancreatic parenchyma by consciously placing the knot on the jejunal side. Moreover, when the pancreas is too thick for the diameter of the jejunum, it is very difficult to perform Blumgart anastomosis. Therefore, Kakita anastomosis may have broader utility. In spite of these minor differences between mattress U-sutures and tangential sutures, the sutures are placed through the full thickness of the pancreas in the same fashion. We believe that both Kakita and Blumgart anastomosis are basically the same method. In addition, blood flow at the pancreatic anastomosis is important to optimize healing of the pancreatic reconstruction [19], and our results indicate that even in Blumgart anastomosis with mattress U-sutures, the rate of latent PF due to ischemia was not higher than that in Kakita anastomosis, as was reported previously [20].

Various strategies to reduce the occurrence and morbidity of postoperative PF are required for optimal outcomes in high-risk patients. The rate of postoperative PF cannot be reduced to zero, especially in patients with a soft pancreas. Previous reports have indicated that it would be possible to abandon routine prophylactic drainage tube placement after pancreatic resection [21–23]. Another prospective randomized controlled multicenter trial strongly demonstrated that routine placement of an intraperitoneal drainage tube in patients undergoing pancreatoduodenectomy reduces the mortality rate [24]. Intraperitoneal drains are routinely placed in our institute. When postoperative PF was evident, the drainage tube was exchanged and maintained with regular exchange until the drainage fluid was nearly absent. Although our method of drainage tube management increased the rate of Grade B PF due to prolonged drain placement, extension of the drain placement duration to avoid intra-abdominal fluid collection did not induce clinically relevant problems, as demonstrated by our low mortality and readmission rates compared with previous reports [1, 2, 25–27]. Our CR-PF rate in patients with a soft pancreas (42.7%) was relatively higher than that in previous reports restricted to high-risk cohorts [17, 27]. However, nearly half of CR-PF cases resulted from extension of the drain placement duration, and no patients developed fever or abdominal pain. Our strategy seems too heterodox and more wasteful than the Western style of early drain removal followed by early discharge. However, we have demonstrated lower mortality and readmission rates than those reported in Western countries, even in an exclusive cohort of patients with a soft pancreas. Although further investigation and validation would be needed to optimize the indication for our drainage tube management in patients with a soft pancreas cohort, our strategy is a promising choice for significantly high-risk patients.

This study does have limitations. First, although the sample size was considerably large, this was a single-institution retrospective study with several operators. However, this study was the largest-scale analysis to date restricted to patients with a soft pancreas who had a high risk of CR-PF. In such a situation, which is similar to the practical setting of each hospital, we have achieved a low mortality rate in high-risk cohorts for postoperative PF. Second, texture of the pancreas was subjective parameter, and potential selection bias could not be eliminated. Third, Kakita anastomosis was our original method, and we were therefore familiar with it. Conversely, Blumgart anastomosis was a new procedure for us. Therefore, our results should be carefully interpreted, considering the difference in the learning curve between the two methods. A large-scale prospective randomized trial is warranted to determine the superiority of the two techniques.

## Conclusions

In conclusion, there was no significance difference in the CR-PF rate between patients who underwent Kakita versus Blumgart anastomosis. Regardless of the anastomosis technique, an accurate and meticulous procedure is essential to achieve a low rate of postoperative PF.

## Abbreviations

CR-PF: Clinically relevant pancreatic fistula
PF: Pancreatic fistula
PJ: Pancreatojejunostomy
